# Osteosarcoma Treated with Pyoktanin

**Published:** 1894-09

**Authors:** J. I. Darby

**Affiliations:** Americus, Ga.


### OSTEOSARCOMA TREATED WITH PYOKTANIN.

By J. I. DARBY, M.D., Am ricus, Ga.

About the 1st of November, 1893, J. J. H., a twelve year old boy of this city, presented himself at my office with a considerable tumor of the left inferior maxillary, which after careful examination was diagnosed osteosarcoma, and as the teeth on this side of the mouth were all loose, he was carried to a dentist and the teeth extracted, after which I gave him a prescription for an antiseptic mouthwash, and requested him to call at my office daily, in order that I might keep the case under observation until a final conclusion was reached upon the subject of operation, as that at first thought appeared to be the only means which suggested itself for his relief. The wash was liberally used as directed, and the boy came every day to have his jaw inspected, which rapidly grew worse as the tumor filled the oral cavity and invaded new bony structure, until it was apparent to the most casual observer that something of a decided nature had to be done for his relief, as he would soon be unable to take food enough to sustain him. Recognizing the great mutilation necessary in a surgical operation for its removal and the proneness of such neoplasm to return, I hesitated to use the knife without first trying the hypodermic injection of pyoktanin, which I had seen suggested as a valuable remedy in similar cases, and accordingly procured a 1 to 500 solution of pyoktanin, made by Merck & Co. of New York, and began its use by daily injections well down into the tumor of about ten drops, gradually increasing the quantity used after the first week up to an ordinary hypodermic syringe half-ful each time, which was kept up this way for near one month, and then injected only every second day, as it was quite plain to be seen that we were getting the disease under control. There was no manifest systemic effect of the drug at any time, but the soft growths very soon showed signs of atrophying and ceased to invade new structures, which, as time went on and the remedy was persevered in, took on healthy action and satisfied us that our efforts would most likely be crowned with success. The treatment was kept up for three months, at the end of which time our patient was discharged cured of his sarcoma and remains in perfect health, with no symptoms of a return of the trouble.

Owing to the great mutilation necessary in removal of the maxillæ by the knife and the proneness of growths of this character to return, I think any reasonable method of treatment, which has nothing worse in it than the possible element of failure, might, with propriety, be resorted to before using the knife. But it is in inoperable cases of sarcoma in young subjects, whose tissues are soft and capable of absorbing the solution of pyoktanin, that I would recommend its use. In order to get the desired result, the remedy must be kept up religiously for quite a while and the patient carefully watched to prevent a return. This treatment, when properly carried out, will, in my opinion, cure as many cases as any other and has the advantage of not being dangerous, which cannot always be said of erysipelatous inoculation and surgical operations. But it is especially in inoperable cases that I would recommend a trial of this remedy.

---

TREATMENT OF THROMBOTIC PILES.—Dr. Bolton Sterns (*Therapeutische Monatshefte*) holds that Fowler's solution causes rapid diminution in the size of thrombotic piles, and finally produces a cure. Sulphur is a still more powerful medicament, not only because of its laxative effect, but from its constitutional influence. The preparation administered was made up of a 1 to 3000 soltion of sulphide of potassium. Of this a teaspoonful was placed in a glass of water, and the latter was taken during the course of the day.

---

HAIR TONIC.—The *Texas Health Journal* says an excellent hair tonic may be made by scalding two ounces of Oolong tea in a gallon of boiling water, straining, and adding three ounces of glycerin, four drachms tincture of cantharides, and one quart of bay rum. After mixing, perfume to suit.

